# Successful mammalian target of rapamycin inhibitor maintenance therapy following induction chemotherapy with gemcitabine and doxorubicin for metastatic sarcomatoid renal cell carcinoma

**DOI:** 10.3892/ol.2014.2118

**Published:** 2014-05-07

**Authors:** KAZUYUKI NUMAKURA, NORIHIKO TSUCHIYA, SUSUMU AKIHAMA, TAKAMITSU INOUE, SHINTARO NARITA, MINGGUO HUANG, SHIGERU SATOH, TOMONORI HABUCHI

**Affiliations:** Department of Urology, Akita University Graduate School of Medicine, Akita 010-8543, Japan

**Keywords:** sarcomatoid renal cell carcinoma, chemotherapy, mammalian target of rapamycin inhibitor

## Abstract

This study presents a case of metastatic sarcomatoid renal cell carcinoma (RCC) treated with systemic chemotherapy followed by mammalian target of rapamycin inhibitor maintenance therapy. A 63-year-old male presented with lumbago, and lumbar vertebral tumors were detected by magnetic resonance imaging. Subsequent computed tomography (CT) revealed a right renal tumor and CT-guided biopsy of the right renal and left sacroiliac tumors determined pure sarcomatoid carcinoma without a clear cell component. Two cycles of combination chemotherapy comprising of gemcitabine (1,500 mg/m^2^ on day one) and doxorubicin (50 mg/m^2^ on day one) resulted in a 20% reduction in the longest diameter of the right renal tumor. However, due to grade 3 neutropenia, the chemotherapy was discontinued and temsirolimus (25 mg once weekly), which binds to the cytoplasmic protein, FKBP-12, and inhibits mTOR, was administered. Stable disease was maintained for 19 months with temsirolimus and no major adverse events, with the exception of grade 2 nausea, were observed. The patient succumbed to their disease at 30 months following the initiation of treatment. These results suggested that systemic chemotherapy followed by temsirolimus maintenance is a feasible treatment option for patients with metastatic sarcomatoid RCC.

## Introduction

Renal cell carcinoma (RCC) accounts for ~3% of new cancer cases and at the time of diagnosis 20–30% of all RCC patients have a metastatic disease (1). The mortality of more than 100,000 patients is due to RCC every year, worldwide (2). Patients with sarcomatoid RCC, which represents a histological variant found in 5–8% of all RCC, have a poor prognosis, with a reported median survival of between nine and 19 months (3). Although several studies with small numbers of patients (<20) have demonstrated the efficacy of chemotherapy (4) and molecular-targeted agents against sarcomatoid RCC (5), no consensus has been reached with regard to the optimal approach for managing sarcomatoid RCC. The current study presents a case of metastatic sarcomatoid RCC treated with systemic chemotherapy followed by mammalian target of rapamycin (mTOR) inhibitor maintenance therapy. Consent was obtained from the family of the patient.

## Case report

**Figure 1:**
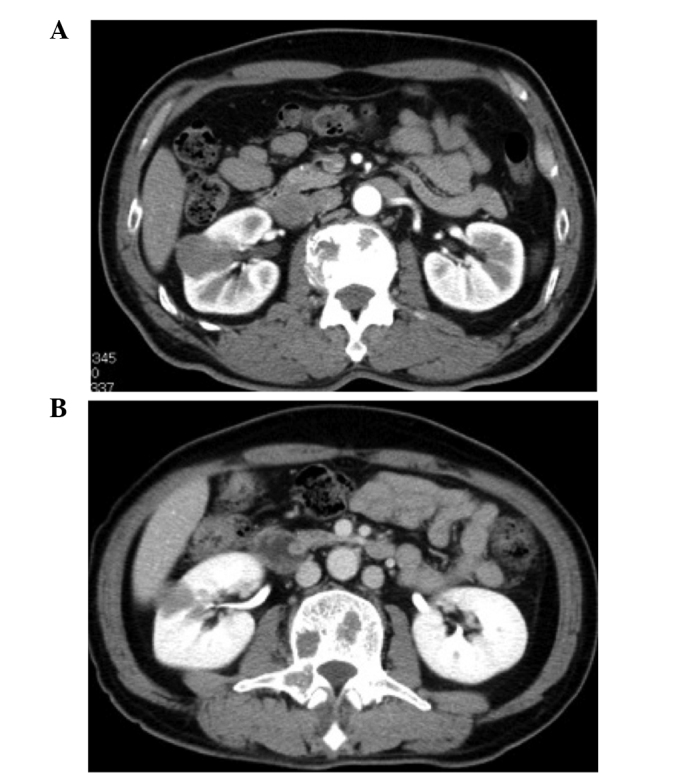
(A) Pretreatment CT revealed a right renal tumor measuring 3.5 cm in diameter. (B) Post-treatment CT demonstrated tumor shrinkage in the right kidney following combination chemotherapy with gemcitabine and doxorubicin. CT, computed tomography.

**Figure 2:**
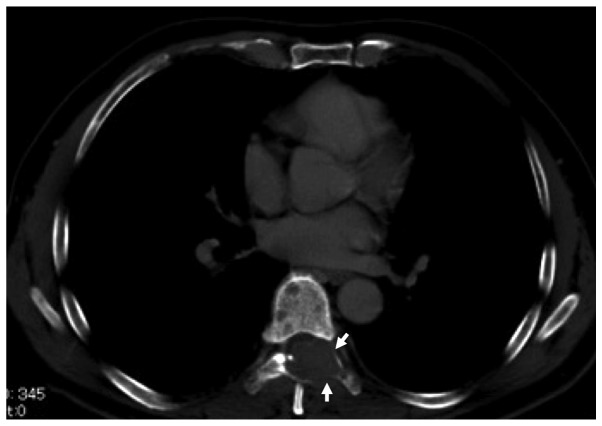
Pretreatment computed tomography revealed a metastatic bone tumor in the ninth thoracic vertebral arch.

**Figure 3:**
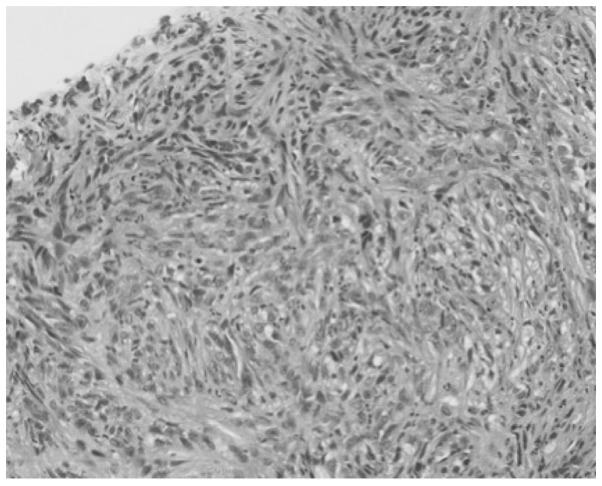
Hematoxylin and eosin staining of a biopsy specimen obtained from the right renal tumor (magnification, ×200).

A 63-year-old male presented with lumbago, and lumbar vertebral tumors were detected on magnetic resonance imaging. Subsequent computed tomography (CT) revealed a right renal tumor and multiple bone metastases (Figs. 1A and 2). CT-guided biopsy of the right renal and left sacroiliac tumors revealed pure sarcomatoid carcinoma without any clear cell component, which was consistent with sarcomatoid RCC and bone metastases (Fig. 3). Radiation therapy (total dose of 30 Gy) was administered to the thoracic vertebrae to relieve the pain due to bone metastases. Two cycles of combination chemotherapy comprising of gemcitabine (1,500 mg/m^2^ on day one) and doxorubicin (50 mg/m^2^ on day one) (4) were administered, resulting in a 20% reduction in the longest diameter of the right renal tumor (Fig. 1B). However, due to grade 3 neutropenia, chemotherapy was discontinued and temsirolimus (25 mg once weekly) was administered, resulting in stable disease for 19 months. During the 21-month treatment with temsirolimus, the patient experienced no major adverse events with the exception of grade 2 nausea. The patient succumbed to their disease 30 months following the initiation of treatment.

## Discussion

Sarcomatoid RCC, which accounts for 4% of all RCC, has an aggressive nature and poor prognosis. Although novel therapies include vascular endothelial growth factor, tyrosine kinase inhibitors and mammalian target of rapamycin inhibitors, all of which have shown significant activity against RCC with metastases, no treatment has been established for sarcomatoid RCC and few studies have analyzed this histological variant. Furthermore, reports discussing the effects of vascular endothelial growth factor-targeted agents are limited. Certain authors have reported the efficacy of tyrosine kinase inhibitors for sarcomatoid RCC only in patients with a limited amount of sarcomatoid components (<20%) in the primary tumor (5). In addition, a recent study from the MD Anderson Cancer Center illustrated that sunitinib shows no benefit in RCC patients with >50% sarcomatoid components (6).

In 2004, Nanus *et al* (7) reported antitumor activity in several patients with sarcomatoid RCC who had been treated with a combination of gemcitabine and doxorubicin chemotherapy. A favorable result led to an Eastern Cooperative Oncology Group phase I trial to confirm the efficacy of this regimen (4). In this study, six of the 39 patients (16%) achieved a complete or partial response and 10 patients (26%) had stable disease; however, 14 patients (37%) developed grade 3 or higher toxicities (4).

Experience with novel targeted agents in sarcomatoid RCC is limited, whereas a phase III trial on temsirolimus versus interferon showed survival benefit for patients with poor prognostic features; likely to be sarcomatoid RCC (8). In addition, a case series by Areses *et al* (9) indicated that toxicity is not the main issue during temsirolimus use for sarcomatoid RCC. Temsirolimus may be a valid therapeutic option with tolerable toxicity for stabilizing tumor progression in sarcomatoid RCC.

Recently, Staehler *et al* (10) reported that combination chemotherapy using gemcitabine and doxorubicin with subsequent antiangiogenic treatment using the multi-tyrosine kinase inhibitor, sorafenib, resulted in additional progression-free survival in five of the nine patients with sarcomatoid RCC. Furthermore, clinical trials on combination therapy comprising of gemcitabine and molecular-targeted agents are ongoing (11).

Immunohistochemistry has shown that sarcomatoid RCC overexpresses hypoxia-inducible factor 1a (HIF-1a) and phosphorylated eukaryotic initiation factor 4E-binding protein (p-4E-BP1) (5). HIF-1a is directly downstream of mTOR, whereas p-4E-BP1 is a direct target of mTOR (12). These results may support the clinical efficacy of mTOR inhibitor in sarcomatoid RCC, which may compensate for the sequential use of chemotherapy and prolong tumor control. Further investigation of the molecular biology of the sarcomatoid variant may elucidate the specific features of sarcomatoid RCC and provide appropriate treatment strategies.
